# Effects of Bi^3+^ Doping on the Optical and Electric-Induced Light Scattering Performance of PLZT (8.0/69/31) Transparent Ceramics

**DOI:** 10.3390/ma12091437

**Published:** 2019-05-02

**Authors:** Bin Zhu, Xia Zeng, Pingsun Qiu, Liang Ling, Dazhi Sun, Suchuan Zhao, Xiyun He

**Affiliations:** 1 Department of Chemistry, College of Sciences, Shanghai University, 99 Shangda Road, Shanghai 200444, China; zhubin0328@163.com; 2 Key Laboratory of Transparent Opto-Functional Inorganic Materials, Shanghai Institute of Ceramics, Chinese Academy of Sciences, 1295 Dingxi Road, Shanghai 200050, China; zengxia@mail.sic.ac.cn (X.Z.); psqiu@mail.sic.ac.cn (P.Q.); lingliang@mail.sic.ac.cn (L.L.); 3 Suzhou Research Institute, Shanghai Institute of Ceramics, Chinese Academy of Science, 6 Liangfu Road, Taicang 215400, China; 4 Key Laboratory of Resource Chemistry of Education Ministry, Department of Chemistry Laboratory, Shanghai Normal University, 100 Guilin Road, Shanghai 200234, China; sundazhi@shnu.edu.cn; 5 Department of Physics, College of Sciences, Shanghai University, 99 Shangda Road, Shanghai 200444, China

**Keywords:** PLZT, electric-induced light scattering behavior, Bi^3+^ ion doping

## Abstract

Lanthanum modified lead zirconate titanate (abbreviated as PLZT) Electro-Optical ceramics with various Bi concentration were prepared by a hot-press process. The PLZT ceramic samples all present a single perovskite structure with no second phase detected. Bi^3+^ ion was considered to mainly enter the A-site of perovskite ABO_3_ structure of PLZT ceramics by the X-Ray Diffractionanalysis. The maximum permittivity (*ε*_m_) of the PLZT samples decreased obviously as Bi concentration increased, while their remnant polarization (*P*_r_) decreased slightly. In particular, the PLZT sample with 0.14 wt% Bi doping presented a higher optical transmittance 61.8% (*λ* = 633 nm), and its electric-induced light scattering performance was found to be improved obviously, the maximum light deduction value of ~47.6% was obtained.

## 1. Introduction

Lanthanum modified lead zirconate-titanate electro-optical (EO) ceramics Pb_1−*x*_La*_x_*(Zr_1−*y*_Ti*_y_*)_1−*x*/4_O_3_, abbreviated as PLZT(*x*/(1−*y*)/*y*), were first reported in 1970 by Haertling [1]. Recently, as the laser modulating technology has been developing rapidly, the PLZT EO ceramics have received a great deal of attention due to their excellent transparency and various EO properties in the range of invisible to near infrared wavelength [2,3,4]. In particular, the PLZT ceramics with a definite composition present reversible electric-induced light scattering properties, which promise important potential applications in high speed EO modulators independent of polarized light [5,6]. When applying an appropriate electric field to these PLZT transparent ceramics, a light scattering phenomenon takes place and the ceramic sample becomes opaque; and when removing this electric field, the light scattering phenomenon will disappear immediately and the ceramic sample reverts to being transparent simultaneously [7,8]. These materials can be used to design and produce the key EO elements applying in various high-speed optical modulators without the polarization system, such as optical attenuators, optical shutters and optical switches, etc. So, it is urgent to optimize and strengthen the electric-induced light scattering properties of PLZT EO ceramics.

A lot of work has been conducted to explore the influences of the material compositions on the ferroelectric, dielectric, and EO properties of PLZT ceramics [9,10,11,12], e.g., the effects of temperature on the PLZT electric-induced light scattering performance [8]. Doping using a few suitable ions is considered as an effective method to modify the material ferroelectric behaviors, which are thought to actually determine the material EO effect [13,14,15]. For example, Bi^3+^ ions doping has been demonstrated to improve the dielectric property of PLZT ceramics [16]. However, little work has been reported about the influence of ions doping on the PLZT electric-induced light scattering properties until now. So, it is meaningful to study the electric-induced light scattering performance of PLZT EO ceramics by doping, and to figure out the relationship between doping ions and material light scattering performance.

In this study, Bi-doping PLZT transparent EO ceramic samples were sintered by a hot-press process. The effects of Bi dopant on the material structures, dielectric and ferroelectric properties, and optical transmittance were measured and discussed systematically. The effects of Bi dopant on the electric-induced light scattering properties of ceramic samples were examined and analyzed.

## 2. Experimental Procedure

In this study, PLZT transparent ceramics with the compositions of a general formula Pb_0.92_La_0.08_(Zr_0.69_Ti_0.31_)_0.98_O_3_ (abbreviated as PLZT (8.0/69/31)) doped with *x* wt% Bi_2_O_3_ (*x* = 0, 0.14, 0.28, 0.42) were yielded.

The starting materials were selected from high purity oxides powders. PbO (Alfa, Montgomery, AL, USA, 99.99%), TiO_2_ (Alfa, 99.99%), ZrO_2_ (Alfa, 99.8%), La_2_O_3_ (Alfa, 99.46%), and Bi_2_O_3_ (Sinopharm, Shanghai, China, 99%) were used to prepare the PLZT samples. The aforementioned materials were added by a weighted calculation, followed by mixing the powders with a planetary ball milling for 4 h in ethanol. Then, the mixtures were dried, mixed with 0.3 wt% of polyvinyl alcohol (PVA, Alfa) and were cold pressed into pellets with a diameter of 35 mm. The pellets contained with different Bi doping were sintered with oxygen atmosphere and an axial press about 16 MPa at 1250 °C for 16 h.

The phase structures of PLZT ceramics were measured by XRD diffraction (XRD, UltimaIV Rigaku, Tokyo, Japan) and Raman (DXR Raman Microscope, Thermo Nicolet, Waltham, MA, USA). The X-ray diffraction measurements were made at 40 kV and 40 mA in 20–80° 2*θ* range, with a step size of 0.02° and 4°/min. The Raman spectra were obtained with a 532 nm solid state laser, which provides characteristic light intensity (6 mW) at the surfaces of samples. The ferroelectric curves of ceramic samples with a thickness of 1.0 mm were carried out in a Work Station equipment (Radiant Technologies, Albuquerque, NM, USA) at room temperature, which were measured at 3500 V. The dielectric properties of PLZT ceramic samples (1 mm thickness) were measured by an E4990A Impedance Analyzer (Keysight Technology, Santa Rosa, CA, USA), with the range of 25–300 °C and the rate of 2 °C/min at the frequencies of 100 Hz, 1 kHz, 10 kHz, 100 kHz, and 1 MHz respectively. The transparency measurement of Bi-doping PLZT ceramic samples with a thickness of 0.50 mm were accomplished by a U-2800 spectrophotometer (Hitachi, Tokyo, Japan) with the wavelength between 200–1100 nm (200 nm/min). The light scattering performances were examined at room temperature by observing the sample transmittance changes under applied electric filed using a He-Ne laser (*λ* = 633 nm) emitter and PM 100D Optical Power Meter (Thorlabs, Newton, NJ, USA).

## 3. Results and Discussion

### 3.1. Structures

**Figure 1:**
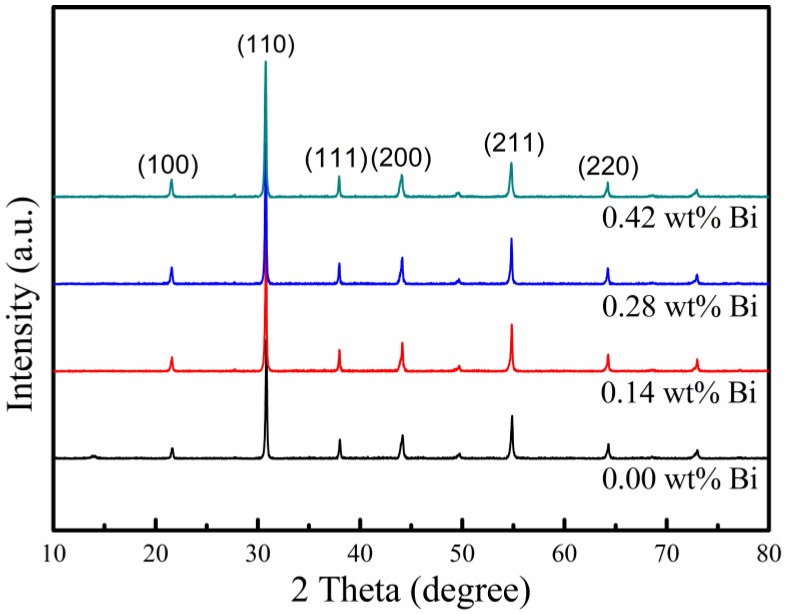
The XRD patterns of Bi doping PLZT transparent ceramic samples.

The XRD patterns of Bi doping PLZT EO ceramics (*x* = 0, 0.14 wt%, 0.28 wt%, 0.42 wt%) were measured and shown in Figure 1. The pure perovskite phase could be clearly observed without any secondary phase. As shown in Table 1, with the Bi concentration increasing, the unit cell volume becomes bigger. It indicates that Bi^3+^ ions entered the crystal lattice and led to the lattice distortion. The radius of Bi^3+^ ions (1.45 Å) is approximate with A-site ions Pb^2+^ (1.49 Å) and La^3+^ (1.36 Å), and much bigger than B-site ions Zr^4+^ (0.72 Å) and Ti^4+^ (0.605 Å) [17], as shown in Table 2. It means Bi^3+^ couldn’t enter the B-site because of the radius of the ions. These facts imply that the Bi^3+^ ions entered the A-site of ABO_3_ perovskite structure of the PLZT material [16].

**Figure 2:**
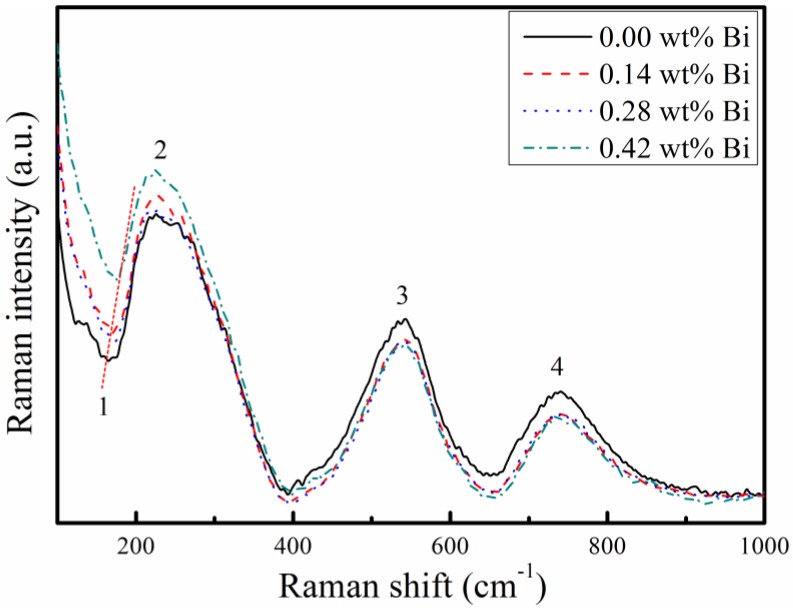
The Raman spectra of PLZT ceramics with Bi-doping. It shows mode 1 (the dip at 170 cm^−1^), mode 2 (a board peak at 227 cm^−1^), mode 3 (a board peak at 542 cm^−1^), and mode 4 (a board peak at 737 cm^−1^).

The Raman spectra of PLZT samples were examined and shown in Figure 2. It has been reported that the modes of perovskite ABO_3_ ceramics were assigned to the phase. Some modes were similar to the modes in Figure 2, including the interference dip at 170 cm^−1^, a broad peak at 242 cm^−1^, a broad peak at 523 cm^−1^, and 724 cm^−1^ [18]. From Figure 2, it can be found that all samples present a perovskite structure.

**Figure 3:**
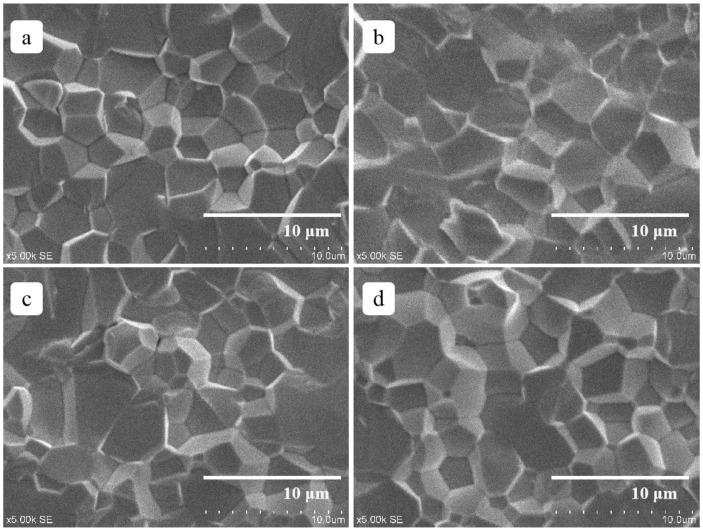
SEM images of different Bi-content doping PLZT ceramics: (**a**) *x* = 0.00 wt% Bi, (**b**) *x* = 0.14 wt% Bi, (**c**) *x* = 0.28 wt% Bi, (**d**) *x* = 0.42 wt% Bi.

Figure 3 shows the SEM images of Bi-doping PLZT transparent ceramics samples. All samples exhibited a uniform and fully dense microstructure, with a little section of transgranular fractures. The average grain sizes of the ceramic sample, examined by the linear intercept method, were 3.26 μm, 3.52 μm, 3.31 μm, and 3.08 μm respectively as Bi concentration increasing. Compared with undoped PLZT ceramics, PLZT ceramic with 0.14 wt% Bi doping enhanced the grain boundary migration, leading to the growth of grain. But as Bi concentration increased, the grain sizes decreased. The possible explanation for this might be that a higher concentration of Bi^3+^ ions near the grain boundaries may restrict the grain growth [19,20].

### 3.2. Dielectric and Ferroelectric Properties

**Figure 4:**
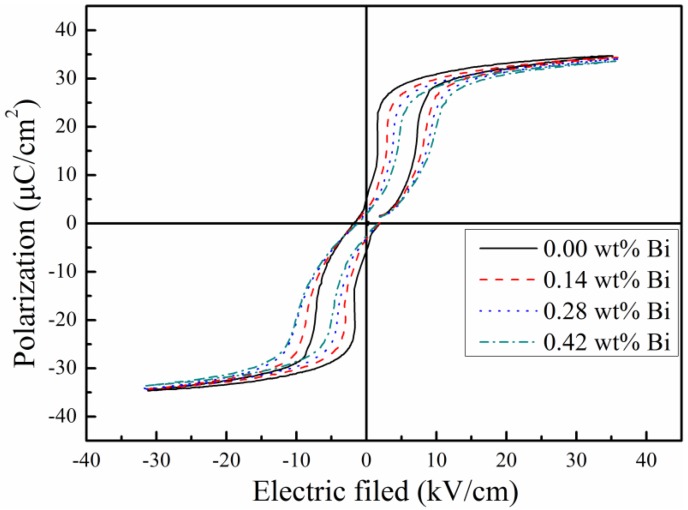
The ferroelectric hysteresis (*P-E*) loops of Bi doping PLZT transparent ceramics.

The ferroelectric hysteresis loops of Bi doping PLZT transparent ceramics were shown in Figure 4. The typical anti-ferroelectric (AFE) phases were observed. The measurement results of remnant polarization (*P*_r_), saturated polarization (*P*_s_), and coercive field (*E*_c_) are all decreased with more Bi^3+^ ions doping, see Table 3. The polarization value and coercive field decreased slightly as the Bi concentration increased. The result was explained by the fact that the modification of Bi-modified for Pb^2+^ at A-site could reduce the high polarizability of PLZT ceramics [21].

**Figure 5:**
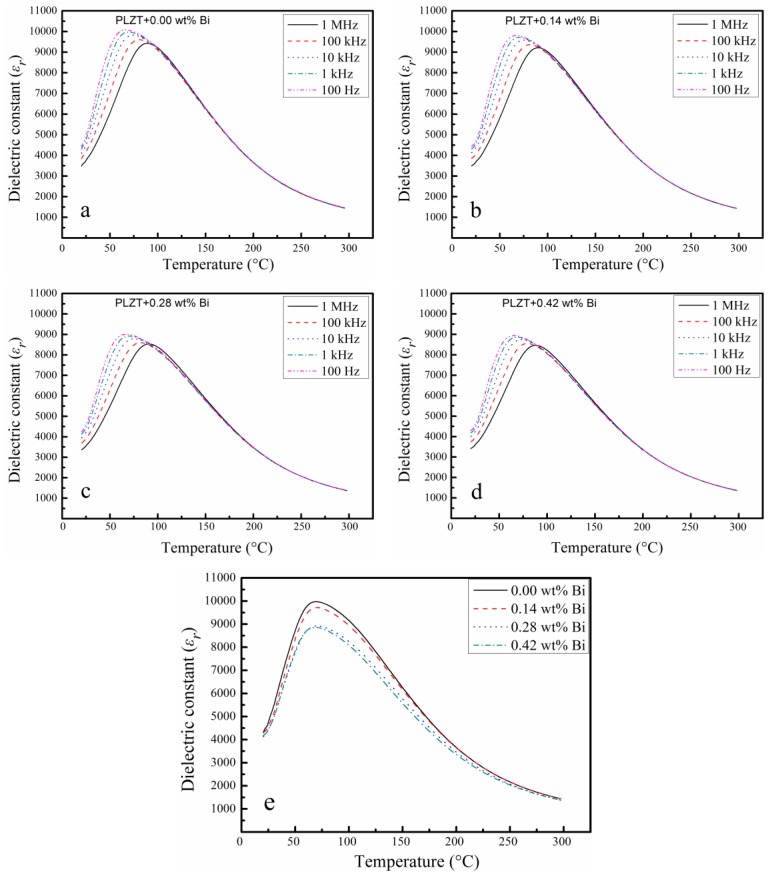
The relative permittivity (*ε*_r_) for Bi-doping PLZT Electro-Optical ceramics as (**a**–**d**) functions of temperature at 100 Hz, 1 kHz, 10 kHz, 100 kHz, 1 MHz respectively; (**e**) a function with different Bi-doping composition at 1 kHz frequency.

Figure 5 shows the temperature dependence of relative permittivity of Bi doping PLZT ceramics at various frequencies. All the samples in this study exhibited typical relaxor ferroelectric characteristics: broad frequency dispersion nearby as well as the temperature (*T*_max_) corresponding to the maximum permittivity. With Bi concentration increasing, the maximum permittivity (*ε*_max_) was decreased, see Figure 5e. The possible explanation for this might be that the vacancy in the unit cell appeared to maintain the electric neutrality while Bi^3+^ ion occupied the A-site. This weakened the coupling of oxygen octahedral established by A-site ions, and further resulted in the dielectric permittivity decreasing.

### 3.3. Optical Transmittance and Electric-Induced Light Scattering Properties

**Figure 6:**
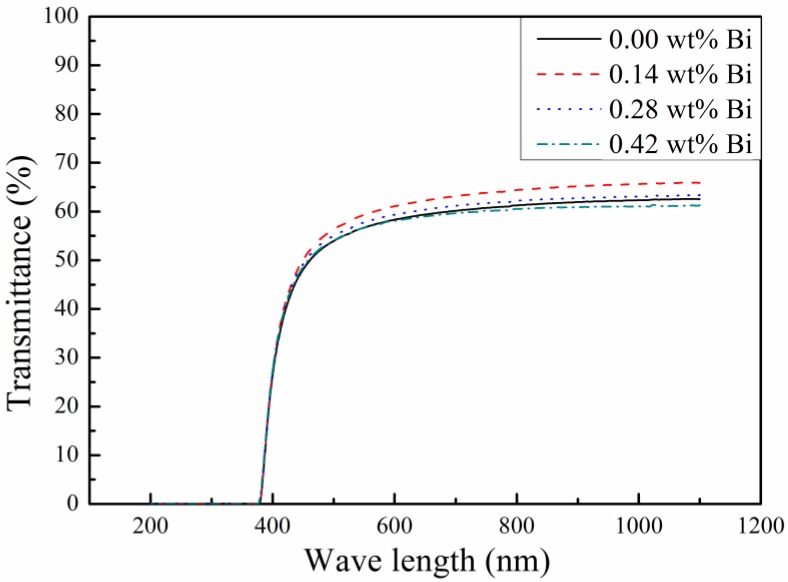
Transmittance spectrum of Bi-doping PLZT transparent ceramics.

The optical transmittance spectrum of Bi doping PLZT transparent ceramic samples with a thickness of 0.50 mm was shown in Figure 6. For PLZT transparent ceramics, the refractive index *n* is 2.48 at 632.8 nm wavelength, and the optical reflection losses resulting from three-grade air/ceramic interface reflection has been reported to be about 30.22% (*λ* = 632.8 nm) [11]. Therefore, the theoretical transmittance rate of 69% of PLZT ceramics can be derived. It can be observed that all the samples present high transmittance close to the theoretical transmittance level, and the transmittance of the PLZT ceramics with 0.14 wt% Bi doping were improved slightly, see Figure 6. While the Bi concentration increasing further, the sample transmittance decreased. This phenomenon can be explained by the possibility that small Bi concentration might increase the *c*/*a* ratio of lattice and enhance the cubic degree, leading to higher transmittance. However, more Bi^3+^ ions accumulated at the grain boundaries and restricted the grain growth with Bi concentration increasing further [20], and the severer light scattering from the more grain boundaries decreased the sample transmittance.

**Figure 7:**
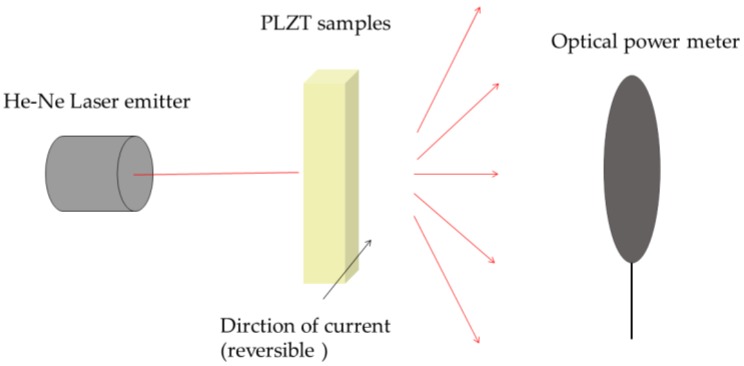
The sketch of measurement of electric-induced light scattering performance.

**Figure 8:**
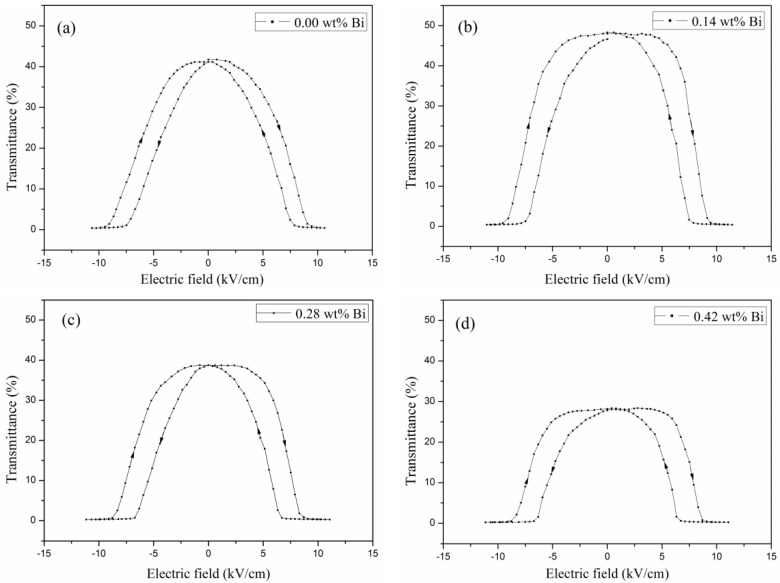
Transmittance versus electric field curves for PLZT ceramics: (**a**) *x* = 0.00 wt% Bi, (**b**) *x* = 0.14 wt% Bi, (**c**) *x* = 0.28 wt% Bi, (**d**) *x* = 0.42 wt% Bi.

In previous works, it was reported that the PLZT electric-induced light scattering performance could be observed as microdomains grew [8,22]. With the suitable size of microdomains, the domain walls would highly influence the light scattering performance. The existence of domain walls would also lower the transmittance of ceramic samples. Figure 7 shows the sketch of measurement of PLZT electric-induced light scattering performance. Figure 8 shows the evolution of transmittance with a changed electric field of PLZT (8.0/69/31) samples with a different Bi concentration at 25 °C. The curves expressed the transmittance variation of PLZT ceramic samples with a variable electric field. All samples were transparent without an electric field and then became opaque for the extensive light scattering effect while a suitable electric field (≥8 kV/cm) was being applied. When the field was removed, the sample transmittance could be recovered. But for the ferroelectric hysteresis property of the PLZT material, a typical hysteresis transmittance curve of the sample was formed. A suitable reversed electric field was found to induce the same light scattering effect. To characterize the light scattering performance concisely, the optical transmittance variation is calculated as the following equation:

*ΔT* = *I*_E0_/*I*_0_ − *I*_E_/*I*_0_, (1)

where *I*_E0_, *I*_E_, and *I*_0_ are respectively the light intensity without electric field, the light intensity with electric field, and the light intensity without PLZT samples. As the Bi concentration increased, the values of *ΔT* of the PLZT transparent ceramic samples were 41.3%, 47.6%, 38.4%, and 27.8% respectively (with a sample thickness of 2.0 mm). It was thought that the amount of domain walls would be increased owing to an enhanced value of remnant polarization (*P*_r_) [23]. In this study, while the electric field removed, the *P*_r_ value of the PLZT ceramic sample decreased as the Bi concentration increasing. This variation may cause the sample transmittance to be improved while the electric field is being removed. Combined with the optical transmittance properties in Figure 6, an optimized *ΔT* value of 47.6% has been obtained in the PLZT ceramic sample with 0.14 wt% Bi doping. This promotion of electric-induced light scattering performance would provide the Bi-doped PLZT (8.0/69/31) ceramics with major potential in specific light modulators.

## 4. Conclusions

PLZT (8.0/69/31) EO ceramics with Bi doping were fabricated by a hot-press process for the first time. The XRD analysis confirmed that the Bi^3+^ ions entered the A-site of perovskite ABO_3_ structure of PLZT ceramics. With Bi concentration increasing, the remnant polarization value (*P*_r_), saturated polarization (*P*_s_), coercive field (*E*_c_), and the maximum permittivity (*ε_max_*) became smaller. 0.14 wt% Bi concentration could improve the electric-induced light scattering performance and optical transmittance of PLZT transparent ceramics. The PLZT (8.0/69/31) EO ceramic sample with 0.14 wt% Bi doping presented a higher optical transmittance of 61.8% (*λ* = 633 nm) and the maximum light deduction value of ~47.6%.

## Figures and Tables

**Table 1:** Lattice parameters of Bi-doping PLZT Electro-Optical ceramics.

Bi Concentration *x*	*a* (Å)	*c* (Å)	*c*/*a*	*V* (Å^3^)
0.00 wt%	5.0439	4.9699	0.95833	126.44
0.14 wt%	5.0316	4.9952	0.99277	126.47
0.28 wt%	5.0320	5.0003	0.99370	126.61
0.42 wt%	5.0493	4.9790	0.98608	126.94

**Table 2:** Parameters of elements in Bi doping PLZT ceramics [17].

Physical Parameter	Pb^2+^	La^3+^	Bi^3+^	Zr^4+^	Ti^4+^
Ionic radius (Å)	1.49	1.36	1.45	0.72	0.605
electronegativity	1.8	1.2	2.0	1.4	1.5

**Table 3:** The values about *P*_r_, *P*_s_, and *E*_c_ of Bi-doping PLZT ceramics.

Bi Concentration *x*	*P*_r_ (μC/cm^2^)	*P*_s_ (μC/cm^2^)	*E*_c_ (kV/cm)
0.00 wt%	4.99	34.69	1.84
0.14 wt%	2.64	34.35	1.76
0.28 wt%	2.39	34.13	1.77
0.42 wt%	2.02	33.60	1.61
